# Risk of hospital admission related to scooter trauma injuries: a national emergency room database study

**DOI:** 10.1186/s12873-022-00711-8

**Published:** 2022-09-01

**Authors:** Sergio M. Navarro, Victor R. Vakayil, Rafat H. Solaiman, Evan J. Keil, Matthew W. Cohen, Ellen J. Spartz, Christopher J. Tignanelli, James V. Harmon

**Affiliations:** 1 Department of Surgery, University of Minnesota, 420 Delaware St SE, Minneapolis, MN 55455 USA; 2 Department of Orthopaedics Rheumatology and Musculoskeletal Sciences, University of Oxford Nuffield, Oxford, UK

**Keywords:** Helmets, safety, regulations, Modeling, Injury, Epidemiology

## Abstract

**Background:**

We investigated key risk factors for hospital admission related to powered scooters, which are modes of transportation with increasing accessibility across the United States (US).

**Methods:**

We queried the National Electronic Injury Surveillance System (NEISS) for injuries related to powered scooters, obtaining US population projections of injuries and hospital admissions. We determined mechanism of injury, characterized injury types, and performed multivariate regression analyses to determine factors associated with hospital admission.

**Results:**

One thousand one hundred ninety-one patients sustained electric-motorized scooter (e-scooter) injuries and 10.9% (131) required hospitalization from 2013 to 2018. This extrapolated to a US annual total of 862 (95% CI:745–979) scooter injuries requiring hospitalization, with estimated annual mortality of 6.7 patients per year (95% CI:4.8–8.5). The incidence of hospital admissions increased by an average of 13.1% each year of the study period. Fall (79 [60%]) and motor vehicle collision (33 [25%]) were the most common mechanism. Injury locations included head (44 [34%]), lower extremity (22 [17%]), and lower trunk (16 [12%]). On multivariable analysis, significant factors associated with admission included increased age (OR 1.02, 95% CI:1.01–1.02), torso injuries (OR 6.19, 2.93–13.10), concussion (25.45, 5.88–110.18), fractures (21.98, 7.13–67.66), musculoskeletal injury (6.65, 1.20–36.99), and collision with vehicle (3.343, 2.009–5.562). Scooter speed, seasonality, and gender were not associated with risk of hospitalization.

**Conclusion:**

Our findings show increased hospital admissions and mortality from powered scooter trauma, with fall and motor vehicle collisions as the most common mechanisms resulting in hospitalization. This calls for improved rider safety measures and regulation surrounding vehicular collision scenarios.

## Background

In May 2019 the Center for Disease Control and Prevention (CDC) announced an emerging epidemic related to powered scooter injuries. Such injuries occur across the globe; one study demonstrated that injuries associated with an estimated average cost of $1693 per injury, and incidence rate of 60 per 100,000 trips [1]. Another study has elucidated the cost of orthopedic surgical care in patients with serious injuries, totaling economic costs as high as $19,282 per person [2].

Electric-motorized scooter (e-scooter) usage has gained popularity in both urban and suburban centers over the past decade [3]. The CDC, along with urban centers in Austin and Los Angeles, have studied a rise in injuries due to e-scooters [4–6]. Despite the sudden rise in popularity, regulations and safety protocols have lagged behind [7]. The lack of helmet use and the interactions with vehicles and pedestrians in public walkways and streets may contribute to injuries. Few studies evaluate these injuries at the national level, with fewer evaluating hospitalization rates from such injuries [8, 9]. A recent temporal analysis at the national level evaluated trends related to e-scooter injury but did not evaluate all available risk factors including helmet usage, scooter speed, seasonality, and alcohol usage [5].

Using a United States national emergency room database, we examined key risk factors that predispose e-scooter users to an increased risk of hospitalization based on emergency room admissions from 2013 to 2018. This study describes the incidence and patterns of injuries, as well as highlights the need for physicians, both emergency room physicians and trauma surgeons, to understand the nature and mechanism of most common injuries. This nationwide study will also help inform broader public policy, safety guidelines, and city planning initiatives.

## Methods

We utilized the United States Consumer Product Safety Commission National Electronic Injury Surveillance System (NEISS) database for this study [10]. The NEISS collates data from approximately 100 participating hospitals that have been selected as a probability sample of all 5000+ emergency departments in the wider United States and United States territories and then extrapolates the data using strata-specific weights to generate national estimates. The sampling frame consists of five strata for hospital types based on hospital size and patient demographics. One stratum includes only children’s hospitals, and the remaining four strata are categorized based on emergency department visits: small (1–16,830), medium (16,831-28,150), large (28,151-41,130), and very large (41,131+). We utilized strata- and hospital-specific weights provided by NEISS to project national estimates. The methodology on the estimation of these weights is published elsewhere [11].

The data collected includes a general diagnosis, specific consumer product code, patient demographics, and brief narratives that describe other aspects of the patient visit in a de-identified manner. For each NEISS designated hospital, a specifically trained physician coordinator compiles the data to ensure a nationally standardized data collection. Additionally, NEISS incorporates sample weights and cluster variables to enable variance calculation and confidence interval estimates for data. Reports run through the NEISS provide the coefficient of variation, which is used to calculate the 95% confidence interval of the estimate [10]. This database has previously been used to characterize nationwide trends of consumer products across numerous specialties [8, 9, 12–14].

We queried the NEISS database for visits specifically related to “Scooters/skateboards, powered” (Code 5042). Within the results, only entries containing the word “scooters” were selected to filter out extraneous results and skateboard accidents. We analyzed data for years dating from 2013 to 2018 to capture and evaluate current estimates and trends. We then filtered entries with injuries specifically requiring hospital admission. Emergency room visits due to e-scooter injuries that did not require admission were excluded, as the primary focus was to define the burden of serious injuries and hospital admissions. The incidence, patient demographic characteristics (i.e. age and sex), and injury characteristics (injury location, disposition, injury diagnosis) were collected from the entries meeting our search criteria. This study qualifies as non-human subject research and was exempt from institutional review board approval because the data is derived from a publicly available database offered by the United States Consumer Product Commission.

The patients were grouped into clinically relevant age groups, including toddlers and young children (0–4 years old), children (5–9 years old), adolescents (10–14 years old), young adults (15–19 years old), adults (20–39 years old), middle-aged adults (40–64 years old), and senior adults (65+ years old). To control for variations within a given year, annual data was broken into two groups for trend analysis; data from 2013 to 2015 comprised the first group, while 2016–2018 comprised the second group within the study.

We then conducted a statistical analysis using SPSS Version 23 (IBM, Armonk, NJ, USA). Individual cases were aggregated on a yearly basis to generate annual rates, with national extrapolation at the national level based on NEISS provided risk weights. For descriptive analysis, total number and percentage were reported for categorical variables, mean and standard deviation for continuous parametric variables, and median and interquartile range [IQR] for continuous non-parametric variables. For descriptive analysis, age was examined in different age grouped cohorts.

Our primary outcome of interest was to identify factors that lead to hospital admission following e-scooter related injuries. We stratified patients on hospital admission status and performed a univariate analysis to compare all baseline variables. To identify independent factors associated with our primary outcome of interest, we constructed a multivariate logistic regression model. Our dependent variable was the presence of hospital admission, and as independent covariates we included all variables that had *P* value < 0.1 on univariate analysis. For analysis, age was examined as a continuous variable in this model. We utilized conditional backward selection to determine independent associations. To confirm the validity of our model, we performed appropriate regression diagnostics, including calculating the Hosmer-Lemeshow goodness-of-fit test, testing for outliers, and using classification tables to compare the predicted vs. actual outcomes. On univariate analysis, to compare both patient cohorts, we used the χ2 test and the Fisher’s exact test for categorical variables, the Mann-Whitney U test for nonparametric continuous variables, and the independent-samples *t*-test for parametric continuous variables.

## Results

**Fig. 1:**
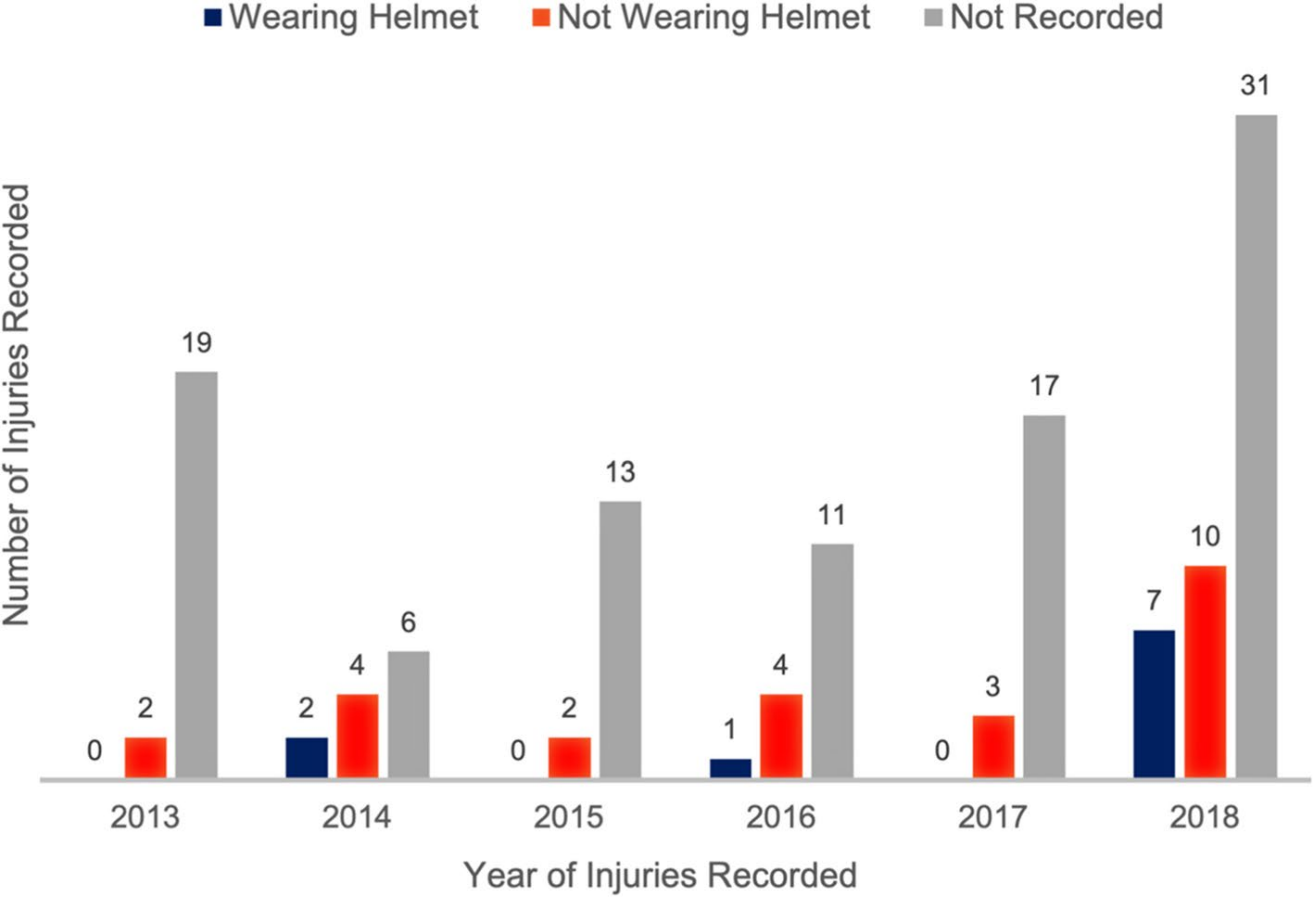
Electric Scooter Injuries Requiring Hospitalization by Year and Helmet Status Between 2013 and 2018

During the study period, a total of 1191 NEISS cases were recorded as e-scooter injuries that occurred in the United States. From 2013 through 2018, we identified 131 (10.9%) electric-motorized scooter injuries requiring hospital admission. Based on NEISS nationwide estimates, this translates to a total of 5173 admissions (95% confidence interval [CI]: 4472 - 5871) secondary to electric-motorized scooter-related trauma nationwide over the time interval [10]. Among the injuries that required admission, the median age of presentation was 32 years (interquartile range: 12–56 years). For adult and elderly age groups, most injuries requiring hospitalization occurred among middle-aged adults between 40 and 64 years old (31/82 [38%]). For those < 20 years old, most injuries requiring admission were among young children aged 5–9 years (23/49 [47%]). When looking at those < 20 years old that were not hospitalized, young children aged 5–9 years comprised only 40% of the group. For males, the average age at admission was 35, while the average age of those not hospitalized was 26. For females, the average age at admission was 40, while the average age of those not hospitalized was 25. Among adults, both young and middle-aged adults were commonly affected while senior adults comprised only 30% of the cohort. The two most common locations for injuries requiring hospitalization were head (44 [33%]) and lower extremities (38 [29%]). The patient’s helmet status was not consistently documented; however, at least 18% of patients presented without helmets (Fig. 1). The overall most common injury was a fracture (61 [47%]) followed by internal organ injury (35 [27%]) (Table 1). The most common sites of injury were the “head” (44 [34%]) and the “ankle” (22 [17%]) (Table 2). The month of June had the highest number of injuries across the time frame (21 [16%]) followed next by August (16 [12%]); however, the month of July only had a total of 8 (6.1%) injuries. January (2 [1.5%]) and February (5 [4%]) had the two fewest number of injuries.

**Table 1:** Electric scooter injuries by diagnosis between 2013 and 2018

Year	Admission Status	Fracture	Internal Organ Injury	Concussion	Contusion	Laceration	Other
**2013**	Yes	8 (40%)	5 (25%)	1 (5%)	1 (5%)	1 (5%)	4 (20%)
	No	20 (13%)	15 (10%)	7 (5%)	52 (34%)	28 (18%)	30 (20%)
**2014**	Yes	2 (15%)	6 (46%)	1 (8%)	1 (8%)	2 (15%)	1 (8%)
	No	25 (23%)	13 (12%)	1 (1%)	28 (25%)	18 (16%)	26 (23%)
**2015**	Yes	8 (53%)	3 (20%)	2 (13%)	1 (7%)	0 (0%)	1 (7%)
	No	31 (24%)	15 (12%)	4 (3%)	33 (27%)	17 (13%)	27 (21%)
**2016**	Yes	6 (38%)	7 (44%)	1 (6%)	1 (6%)	0 (0%)	1 (6%)
	No	32 (22%)	13 (9%)	8 (5%)	39 (26%)	26 (18%)	30 (20%)
**2017**	Yes	11 (55%)	5 (25%)	2 (10%)	2 (10%)	0 (0%)	0 (0%)
	No	48 (26%)	13 (7%)	6 (3%)	39 (21%)	21 (12%)	56 (31%)
**2018**	Yes	26 (65%)	9 (24%)	2 (5%)	1 (2%)	1 (2%)	1 (2%)
	No	76 (22%)	28 (8%)	7 (2%)	85 (24%)	54 (16%)	98 (28%)

**Table 2:** Electric scooter injuries by body part between 2013 and 2018

Year	Admission Status	Head	Ankle	Lower Trunk	Upper Trunk	Upper Leg	Foot	Other
**2013**	Yes	7 (35%)	4 (20%)	2 (10%)	1 (5%)	1 (5%)	1 (5%)	4 (20%)
	No	26 (17%)	7 (5%)	9 (6%)	7 (5%)	1 (1%)	12 (8%)	90 (60%)
**2014**	Yes	6 (50%)	0 (0%)	0 (0%)	1 (8%)	0 (0%)	2 (17%)	3 (25%)
	No	22 (20%)	8 (7%)	3 (3%)	4 (4%)	0 (0%)	11 (10%)	63 (57%)
**2015**	Yes	5 (33%)	1 (7%)	3 (20%)	2 (13%)	0 (0%)	2 (13%)	2 (13%)
	No	21 (16%)	11 (9%)	8 (6%)	2 (2%)	2 (2%)	8 (6%)	75 (59%)
**2016**	Yes	8 (50%)	1 (6%)	3 (18%)	2 (13%)	0 (0%)	0 (0%)	2 (13%)
	No	24 (16%)	15 (10%)	8 (5%)	6 (4%)	2 (1%)	12 (8%)	81 (56%)
**2017**	Yes	7 (41%)	6 (35%)	3 (18%)	0 (0%)	0 (0%)	0 (0%)	1 (6%)
	No	26 (14%)	20 (11%)	3 (2%)	6 (3%)	1 (1%)	10 (5%)	117 (64%)
**2018**	Yes	11 (27%)	10 (24%)	5 (12%)	4 (10%)	5 (12%)	1 (3%)	5 (12%)
	No	44 (13%)	25 (7%)	18 (5%)	8 (2%)	5 (1%)	16 (5%)	232 (67%)

At the start of the study period in 2013 through 2015, the annual incidence of electric-motorized scooter-related injuries requiring hospitalization was estimated to be 790 cases per year (95% CI: 683–896). Over the following period from 2016 through 2018, the annual incidence of electric-motorized scooter-related hospitalization related injuries increased to 1895 cases per year (95% CI: 1639-2151). This extrapolates to an increase of approximately 2.4 times the annual incidence of hospitalizations from electric-motorized scooter trauma from the 2013 to 2015 period. The number of admissions for female riders did not increase significantly during the study period, ranging consistently between four and seven injuries resulting in admission. For males, however, the number of injuries significantly rose from a low of 7 injuries in 2014 to a high of 42 injuries resulting in admission in 2018. Univariable analysis of hospital admission suggested an increase in male e-scooter injuries (*p* = .032), demonstrating a higher risk of admission in the male cohort; however, on multivariable analysis, sex was not identified as an independent indicator of hospital admission. Age, neither increased nor decreased significantly during the study period.

Of the injuries where race was recorded (92/131, 70%), White race comprised the highest number of cases (66/92, 72%) followed by Black or African American (22/92, 24%), and a total of four individuals were either Asian American or Native American. The only substantial increase among any of the racial groups was found within the Black or African American cohort, in which from 2013 to 2017 there were a total of seven injuries resulting in admission, and in 2018 alone there were a total of 14 such injuries. Between the two-year brackets (2013–2015 and 2016–2018), there was an increase of 144% from 27 to 39 injuries in the White cohort compared to an increase of 633% from 3 to 19 injuries resulting in admission in the African American cohort.

Multivariable analysis identified variables independently associated with admission following an e-scooter injury (Tables 3 and 4). Age was provided for all injuries within the NEISS and had an odds ratio of 1.02 (95% CI: 1.01–1.02, *p* < 0.001). Injury to specific body parts was also associated with an increased odds of hospital admission following an e-scooter injury. The torso was found to have an odds ratio of 6.19 (95% CI: 2.93–13.10, *p* < 0.001) when compared to an injury to the upper and lower limbs. Four separate diagnoses were also found to predict hospital admission: concussion, fracture, musculoskeletal injury, and collision with another vehicle. Odds ratios were 25.45 for concussion (95% CI: 5.88–110.18), 21.97 for fracture (95% CI: 7.13–67.67), 6.65 for musculoskeletal injuries (95% CI: 1.20–36.99), and 3.34 for collision with another vehicle (95% CI: 2.01–5.56) when compared to sprains and strains. Patients with injuries who presented to a small hospital compared to large hospitals were less likely to require admission (OR = 0.15, 95% CI: 0.03–0.73, *p* < 0.019). Seasonal analysis of injuries revealed a non-significant difference between the four groups (*p* = 0.129).

**Table 3:** Multivariate analysis of risk factors associated with electric scooter injuries

		Admission Status								
		No				Yes^a^				
		Count	Percentage	Mean	Standard Deviation	Count	Percentage	Mean	Standard Deviation	***P*** value
Total		1095	100%			96	100%			
Day	Weekday	722	65.9%			67	69.8%			0.500
	Weekend	373	34.1%			29	30.2%			
Season	Fall	287	26.2%			17	17.7%			0.129
	Spring	256	23.4%			31	32.3%			
	Summer	371	33.9%			34	35.4%			
	Winter	181	16.5%			14	14.6%			
Age				26	24			37	31	< 0.001
Sex	Male	677	61.8%			72	75.0%			0.011
	Female	418	38.2%			24	25.0%			
Race	Not Recorded	337	30.8%			28	29.2%			0.567
	White	494	45.1%			49	51.0%			
	African American	200	18.3%			16	16.7%			
	Asian American	48	4.4%			1	1.0%			
	Native American	14	1.3%			2	2.1%			
	Pacific Islander	2	0.2%			0	0.0%			
Helmet Use	Yes	44	38.3%			8	38.1%			0.989
	No	71	61.7%			13	61.9%			
Location	Not Recorded	399	36.4%			25	26.0%			0.008
	Home	212	19.4%			13	13.5%			
	Street or Highway	307	28.0%			45	46.9%			
	Public Property	150	13.7%			11	11.5%			
	School	5	0.5%			0	0.0%			
	Recreational	22	2.0%			2	2.1%			
Multiple Riders	No	1075	98.2%			95	99.0%			1.000
	Yes	20	1.8%			1	1.0%			
Thrown from vehicle	No	177	16.2%			15	15.6%			1.000
	Yes	918	83.8%			81	84.4%			
Scooter struck MV^b^	No	943	86.1%			65	67.7%			< 0.001
	Yes	152	13.9%			31	32.3%			
Speed > 20	No	39	69.6%			6	60.0%			0.714
	Yes	17	30.4%			4	40.0%			
Alcohol	No	1077	98.4%			91	94.8%			0.032
	Yes	18	1.6%			5	5.2%			
Region of Body	Head/neck/face	338	30.9%			28	29.2%			< 0.001
	Limbs	655	59.8%			42	43.8%			
	Multiple	8	0.7%			1	1.0%			
	Other	4	0.4%			0	0.0%			
	Torso	90	8.2%			25	26.0%			
Diagnosis	Brain	143	13.1%			23	24.0%			< 0.001
	Burns	8	0.7%			0	0.0%			
	Contusion/laceration/abrasions	447	40.8%			8	8.3%			
	Fracture	236	21.6%			54	56.3%			
	Internal organ	1	0.1%			3	3.1%			
	Musculoskeletal	41	3.7%			3	3.1%			
	Strain/sprain	219	20.0%			5	5.2%			
Stratum	Childrens	202	18.4%			21	21.9%			0.050
	Large	192	17.5%			17	17.7%			
	Medium	178	16.3%			13	13.5%			
	Small	122	11.1%			2	2.1%			
	Very Large	401	36.6%			43	44.8%			

^a^ Only includes patients who were treated and admitted

^b^ MV = motor vehicle

**Table 4:** Descriptive analysis of risk factors for hospitalization from electric scooter injuries between 2013 and 2018

Variable	OR	95% CI		***P*** Value
Age	1.015	1.006	1.023	< 0.001
Body Part^a^				
Torso	6.193	2.929	13.096	< 0.001
Diagnosis^b^				
Concussion	25.451	5.879	110.182	< 0.001
Fracture	21.975	7.125	67.663	< 0.001
Musculoskeletal Injury	6.652	1.196	36.994	0.030
Collision with another Vehicle	3.343	2.009	5.562	< 0.001
Hospital Type^c^				
Small Hospital	0.150	0.030	0.734	0.019

^a^Reference category = Upper and lower limb

^b^Reference category = Strain, sprain and pain

^c^Reference category = Very large hospital

C-Statistic = 0.52, Hosmer-Lemeshow Test *P* = 0.154

## Discussion

Electric scooter injuries are rising globally, in line with increasing e-scooter usage, and our analysis of six-year data identifies similar trends in the United States [15]. Specifically, we observed a near two-fold increase in hospitalization following e-scooter injury. This epidemic of electric-motorized scooter-related traumatic injuries and the associated emergency care consumption represents a rising public health concern. In addition to a rise in ED visits, there are substantial secondary costs including hospitalizations, subspecialty consultations, operations, medications, and follow visits.

Consequently, there should be an active development of preventative strategies. Regarding preventative medicine, our study directly touches upon the key issue of personal protective equipment. In our analysis, the most common injury was a “head” injury (33.6%), and only 29% of cases where helmet status was reported affirmed that patients were wearing a helmet upon arrival to the emergency room. While data was limited, in this cohort, 71% of patients in which helmet status was recorded were not wearing a helmet at the time of injury.

Helmets have repeatedly demonstrated impact mitigation in other types of similar personal transportation vehicles including bicycles and motorcycles [4, 16]. Recognizing this fact, laws for these devices have been adapted to electric-motorized scooters in other countries. For example, in March of 2000, Italy implemented a universal helmet law mandating helmet use for all types of recreational scooter drivers including electric-motorized scooters [17]. After implementation, there was a 66% decrease in traumatic brain injury (TBI) occurrences in motorcycle-moped crashes. This data could be used to support efforts for universal helmet laws in the United States to decrease TBI incidence rates. The state of California recently changed its helmet laws regarding electric electric-motorized scooters to a less strict version, now only requiring those under 18 years old to wear a helmet where previously all riders were required to wear a helmet [7]. The impact of this change ought to be monitored closely as rates of TBI occurrences may increase as helmet usage decreases, as has been shown in motorcycle accidents. There is no standardized legislation regarding the utilization of these scooters or the use of protective equipment. Legislation, where existing, varies markedly based on state and city of legislation. Electric scooter legislation is rapidly evolving to combat their increase in popularity but there is still a significant delay in most parts of the country and uncertainty on how to best handle these issues.

The multivariable analysis revealed factors associated with an increased risk of admission in individuals that presented to an emergency department due to an electric scooter injury. A disparate number of males (42) were admitted in 2018 as compared to females (6), which may be linked to the higher usage of e-scooters, less helmet use, higher risk driving, as well as increased substance abuse or intoxication in males [18, 19]. Presenting to a small hospital was associated with a decreased admission rate (*p* = 0.019), likely highlighting the hierarchy of care in e-scooter related trauma. It is likely that severe cases are directed to larger facilities whereas less severe injuries can be treated at small facilities.

Limitations of this study are inherent to the database utilized. Although carefully designed to utilize a selected 100 hospitals to provide an estimate for injuries on a national level, the participating emergency rooms may be in states with varying regulations regarding electric-motorized scooters or outside urban areas where electric-motorized scooter use may be more common, potentially over- or underestimating the true national incidence. Additionally, a portion of patients with less severe injuries from scooter-related accidents may present elsewhere, such as urgent care centers or to their primary care physician. Also important to address are the well-documented limitations that data on race and ethnicity within medical charts and aggregate national data have. Often the data can be incomplete (nearly one-third of our data did not include racial data) and may rely on observations of the one who filled out the record, as compared to direct interviews. These points, in addition to the limited data set, must be considered when viewing and interpreting these findings [20].

This study, which demonstrates a moderate increase in hospitalization requiring injuries from motorized scooter use over the past half-decade, is a key data point in the discussion regarding electric-motorized scooter use and public health. That said, the e-scooter injuries found in the NEISS database are limited in its accuracy as a specific patient diagnosis code is not available, nor are procedure codes associated with treatment modalities. Also of importance, helmet status was not consistently recorded in the NEISS database, nor was alcohol use. Furthermore, the type of scooter was not specifically outlined, as standing scooters have increased in use in urban areas, whereas electric-motorized sitting scooters have remained constant.

Individualized patient data in a prospective large institutional study would allow detailed analysis of injuries and may offer further insight into the effectiveness of helmets for protection from electric-motorized scooter-related injuries. Identifying the common patterns of injury requiring hospitalization related to specifically electric-motorized scooters will be useful for patient education and injury prevention in the future.

## Conclusion

Electric-motorized scooters are an increasingly popular method of transportation, which coincides with a temporal increase in patients requiring hospitalization for traumatic injuries from electric-motorized scooter accidents. Our findings are consistent with a recent 2019 case series from urban trauma centers and a trend analysis showing increased admission rates from e-scooter trauma. However, our study provides additional insight on the role of alcohol usage and implication of vehicular related incidents associated with e-scooter trauma resulting in hospital admission. Understanding these trends is an important patient safety issue and requires the development of appropriate public policies. As we have examined ridership exposure, the near doubling of incident e-scooter trauma and involvement with motor vehicles calls for improved rider safety measures and regulation, in particular surrounding regulation of collision scenarios, speed, alcohol use, or helmet use. E-scooter companies should continue to facilitate and encourage helmet access. Legislation surrounding motor vehicle interaction with e-scooters and additional research in this area may help mitigate the risk of e-scooter trauma events.

## Data Availability

Available upon request.

## Abbreviations

E-scooters: Electric scooters
ED: Emergency Department
NEISS: National Electronic Injury Surveillance System
SPSS: Statistical Product and Service Solutions
IQR: Interquartile range
US: United States
